# Computed Fluid Dynamics-Based Blood Pressure Prediction for Coronary Artery Disease Diagnosis Using Coronary Computed Tomography Angiography

**DOI:** 10.3390/jimaging12050196

**Published:** 2026-05-02

**Authors:** Rene Lisasi, Huan Huang, William Pei, Michele Esposito, Chen Zhao

**Affiliations:** 1 Department of Computer Science, Kennesaw State University, Marietta, GA 30060, USA; rlisasi@students.kennesaw.edu (R.L.); hhuang10@students.kennesaw.edu (H.H.); wpei@kennesaw.edu (W.P.); 2 Department of Cardiology, Medical University of South Carolina, Charleston, SC 29425, USA; espositm@musc.edu

**Keywords:** coronary artery disease, coronary computed tomography angiography, blood pressure prediction, diffusion, inverted conditional diffusion

## Abstract

Computational fluid dynamics (CFD)-based simulation of coronary blood flow provides valuable hemodynamic markers, such as pressure gradients, for diagnosing coronary artery disease (CAD). However, CFD is computationally expensive, time-consuming, and difficult to integrate into large-scale clinical workflows. These limitations restrict the availability of labeled hemodynamic data for training AI models and hinder the broad adoption of non-invasive, physiology-based CAD assessment. To address these challenges, we develop an end-to-end pipeline that automates coronary geometry extraction from coronary computed tomography angiography (CCTA), streamlines simulation data generation, and enables efficient learning of coronary blood pressure distributions. The pipeline reduces the manual burden associated with traditional CFD workflows while producing consistent training data. Furthermore, we introduce a diffusion-based regression model. Specifically, the inverted conditional diffusion (ICD) model is designed to predict coronary blood pressure directly from CCTA-derived features, thereby bypassing the need for computationally intensive CFD during inference. The proposed model is trained and validated on two CCTA datasets using the Adam optimizer with a weight decay of $1\times 10^{-3}$, a learning rate of $1\times 10^{-5}$, a batch size of 100, and Huber loss. It is then evaluated on a test set of ten simulated coronary hemodynamic cases. Experimental results demonstrate state-of-the-art performance. Compared with Long Short-Term Memory (LSTM), the proposed model improves the $R^{2}$ score by 19.78%, reduces the root mean squared error (RMSE) by 19.44%, and lowers the normalized root mean squared error (NRMSE) by 18%. Compared with a multilayer perceptron (MLP), it improves the $R^{2}$ score by 8.38%, reduces RMSE by 4.3%, and reduces NRMSE by 5.4%. This work represents a first step toward a scalable and accessible framework for rapid, non-invasive, CFD-based blood pressure prediction, with the potential to support CAD diagnosis.

## 1. Introduction

Coronary artery disease (CAD) is characterized by plaque build-up in the coronary arteries, which can restrict blood flow, impair cardiac function, and ultimately lead to the development of heart failure [1]. Non-obstructive CAD can often be managed with medication alone, whereas an obstructive lesion, in which a coronary artery is blocked by ≥70%, may require further treatment. In clinical practice, when a lesion visualized on invasive coronary angiography is indeterminate, the functional significance must be carefully evaluated to determine the appropriate management strategy. Fractional flow reserve (FFR) is a widely used clinical index for assessing the physiological impact of coronary artery stenosis. It is defined as the ratio of maximal blood pressure distal to a stenotic lesion to the maximal aortic pressure under conditions of induced hyperemia. FFR provides a quantitative measure of the extent to which coronary narrowing limits blood flow to the myocardium. An FFR value ≤ 0.80 indicates hemodynamically significant stenosis and reduced myocardial function [2], in where revascularization procedures such as percutaneous coronary intervention (PCI) or coronary artery bypass grafting (CABG) are generally recommended [3].

Clinically, FFR is measured invasively using a pressure-sensing guidewire that is advanced across the lesion during coronary angiography. This wire-based FFR measurement is considered the gold standard for determining whether a stenosis is hemodynamically significant. Despite its clinical reliability, wire-based FFR is invasive [4], time-consuming, and associated with procedural risks and additional costs, as 27.4% of device failures result in patient adverse events [5]. As a result, computational fluid dynamics (CFD)-based blood pressure prediction has emerged as a non-invasive alternative for estimating FFR. By simulating coronary blood flow and pressure proximal and distal to suspected stenotic segments, CFD-based approaches enable preliminary functional assessment without the need for a pressure wire. Therefore, accurate blood pressure prediction is a crucial step toward reliable non-invasive FFR estimation and early identification of CAD.

In recent years, non-invasive FFR estimation has been developed using computational modeling techniques [6]. These approaches do not measure pressure directly but instead compute FFR from anatomical data derived from coronary imaging, and fractional flow reserve computed tomography ($FFR_{CT}$) that is employed in clinical practice [7]. Invasive coronary angiography (ICA), while highly accurate, is invasive and involves an arteriotomy through which a guiding catheter is maneuvered through the vasculature to the aortic root, posing considerable procedural risk [8]. In contrast, coronary computed tomography angiography (CCTA) provides three-dimensional anatomical imaging of the coronary arteries with lower risk, which requires the injection of a contrast agent before a CT scan [7,9]. $FFR_{CT}$ models the coronary artery tree based on CT images and uses CFD to simulate blood flow. Cardiologists can then visualize these simulations to assess the functional significance of stenoses and inform treatment planning, ranging from medication for low-risk cases to surgical intervention for high-risk patients.

Despite its clinical utility, $FFR_{CT}$ relies on complex CFD simulations that involve multiple preprocessing and modeling steps, including image loading, segmentation, geometry reconstruction, meshing, boundary condition assignment, and numerical simulation. Also, transient CFD analysis requires solving complex nonlinear equations with millions of degrees of freedom, typically resulting in computational times of 12–24 h per case [10], which limits its use in emergency settings and rapid clinical decision-making. However, commercial solutions such as HeartFlow [11,12] and ArteryFlow [13] demonstrate the feasibility of CCTA-based functional assessment but remain constrained by the inherent computational burden of CFD workflows.

To address these limitations, data-driven approaches based on deep learning have been explored for direct hemodynamic prediction from imaging data. Conventional regression architectures, including ResNet-based feature extractors [14], multilayer perceptrons (MLPs) [15], and recurrent models such as LSTMs and Bi-LSTMs [16], have been applied to continuous prediction tasks but may suffer from limited robustness when modeling complex spatial pressure distributions. More recently, diffusion models [17], initially developed for image synthesis, have demonstrated potential in medical imaging applications, including conditional CT generation [18]. Compared with approaches such as Classification and Regression Diffusion (CARD) [19], which primarily focus on uncertainty estimation, diffusion-head-based frameworks offer a structured refinement mechanism for discrete and continuous-value prediction. These emerging methods provide a scalable and computationally efficient alternative to simulation-based strategies and may facilitate broader clinical adoption of non-invasive functional assessment from CCTA.

**Figure 1:**
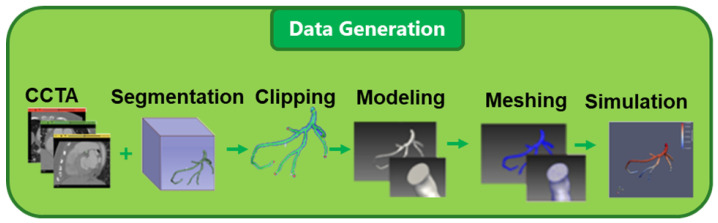
CCTA-based blood pressure prediction. From left to right: CCTA scan, 3D segmentation, clipping, modeling, meshing, and blood flow simulation using SimVascular 2023 [20].

Importantly, FFR is fundamentally defined as a pressure ratio across a coronary stenosis under hyperemic conditions. Therefore, accurate estimation of coronary blood pressure is the key step in FFR computation. Motivated by this observation, this study proposes a deep learning-based framework that leverages CCTA to directly predict coronary pressure distribution, thereby enabling efficient FFR estimation without performing full CFD simulations. Performing CFD simulations from CCTA typically involves multiple steps: (a) loading CT images, (b) loading the 3D artery segmentation, (c) clipping the artery ends, (d) modeling the clipped artery, (e) meshing the model, (f) setting simulation parameters, (g) running the blood flow simulation, and (h) processing the simulation results, as illustrated in Figure 1.

The proposed approach consists of two main components: an automated data pipelining framework and a novel deep learning model designed for hemodynamic prediction. Together, these components enable non-linear estimation of CFD-derived coronary pressure distributions directly from medical imaging data. The data pipeline standardizes and streamlines the generation of datasets by automating key preprocessing steps, including coronary artery segmentation, vessel clipping, transformation of patient-specific geometries into a unified reference coordinate system and application of necessary linear mappings. This structured preprocessing ensures geometric consistency across patients and facilitates robust model training. Building on this pipeline, the proposed deep learning framework is trained to predict blood pressure throughout the coronary artery tree without requiring full CFD simulations. Since FFR is fundamentally derived from pressure ratios, accurate pressure prediction enables an efficient functional assessment of coronary stenosis. By eliminating the need for computationally intensive CFD workflows, the proposed method has the potential to reduce diagnostic time and cost, increase clinical performance, and minimize patient risk associated with invasive evaluation procedures.

## 2. Materials and Methods

The proposed method is made up of two main parts: the Patch-Based Dataset Pipeline (PBDP) and inverted conditional diffusion (ICD) for blood flow prediction. The data pipeline consists of simulating blood flow in coronary arteries and extracting the centerline, image patches, and scalar pressures at each point for every *r* into a dataset. The added improvement to the pipeline is converting the volume to Left Posterior Superior (LPS) coordinate system so that all files share a common coordinate system, then transforming the volume to the center of the pressure polygon so that it aligns the patches with the centerline, before finally converting world voxel coordinates to local coordinates.

### 2.1. Blood Fluid Simulation Using CCTA

The blood fluid data for coronary arteries is generated through a multi-step pipeline involving data loading, artery clipping, centerline extraction, modeling, meshing, parameter assignment, simulation, and postprocessing, as shown in Figure 1. Each step is detailed below.

**Figure 2:**
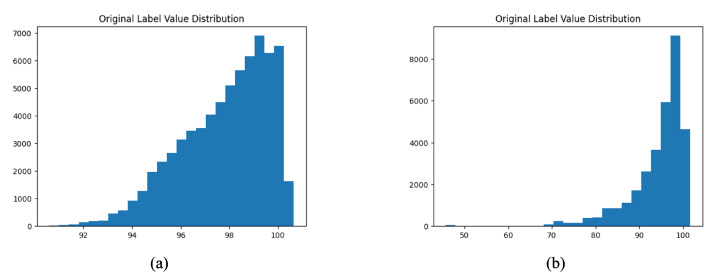
Average pressure distribution (mmHg) across datasets: (**a**) CCTA1000, (**b**) CCTA36.

(1) Data Loading and Artery Clipping. Raw patient data, including CT scans and corresponding 3D segmentation labels, are imported into 3D Slicer [21]. Within the Slicer interface, the Segmentation Editor is selected, and the 3D view is used to visualize the coronary artery. The target artery is then clipped to isolate the region of interest.

(2) Centerline Extraction. Using the VMTK module in Slicer, centerlines are extracted from the segmented arteries. The surface is set to the segmentation label, and new endpoints are defined. A new centerline model is generated and applied, with endpoint adjustments ensuring that endpoints are located only at the termini of the artery tree [22].

(3) Inlet and Outlet Modification. To prepare the artery for CFD meshing, the inlet and outlet segments are clipped using VMTK’s Clip Vessel module in 3D Slicer. The newly generated centerline and endpoints are used to define clipping locations. Caps are optionally generated for the inlet and outlet surfaces; however, final capping can be deferred to the modeling step for precise boundary treatment.

(4) Modeling. Using SimVascular, the clipped artery is imported as a model. Face extraction is performed with a separation angle of $50^{\circ }$, and the global representation is reinitialized. Surfaces not corresponding to anatomical structures are removed, holes are filled, and caps are labeled according to their anatomical location (e.g., left anterior descending, LAD).

(5) Meshing. A new mesh is generated for the imported model. Mesh size is estimated, and the mesher is executed. Successful meshing requires properly clipped inlets and outlets; if meshing fails, the clipping and capping procedures are adjusted. The model can then be re-meshed as necessary to ensure mesh quality. The completed meshes had an average of 62,088 nodes, 321,323 elements, 65,734 edges, and 43,822 faces.

(6) Blood Flow Simulation. The below parameters are assigned to perform the blood flow simulation using SimVascular. Using the configured mesh and parameters, simulations are executed in SimVascular. Parallel computing with MPI is enabled to leverage available computational resources (10 processes in this setup). The simulation generates time-resolved hemodynamic data across 150 timesteps × 0.001 s = 0.15 s of a 0.8 s cardiac cycle. Due to the time-consuming nature of a full cardiac cycle simulation, only 12% of a cardiac cycle’s timesteps were simulated. After performing the simulation, visualization and analysis are conducted in ParaView [23], enabling playback of the entire simulation. Average pressures are extracted using the averaged mmHg field for downstream analysis. The average pressure distributions for datasets CCTA1000 and CCTA36 (introduced in Section 2.7) are shown in Figure 2.

The initial pressure was set to 133,300 Pa, which yielded an output of around 100 mmHg in the reported results. The value 13,330 Pa directly converts to 100 mmHg; however, using it would not yield the expected results. It is possible that Simvascular internally scales down the average pressure in mmHg by a factor of 10. To avoid confusion, we use 133,300 Pa, then during training, we use the “average pressure mmHg” property from the simulation file as the ground truth. For boundary conditions, all outlet caps are assigned a resistance of 1333 Pa·s/cm^3^, except for the inlet. The vessel wall properties are defined as follows: wall thickness of 0.2 mm, elastic modulus of $4\times 10^{6}$ Pa, density of 0.8 g/cm^3^, and wall pressure of 133,300 Pa [24]. The remaining solver parameters are summarized in Table 1 [25]. Simvascular adopts laminar flow modeling and no turbulence model. The blood viscosity is 0.04 g/cm × s^2^ and blood density is 1.06 g/cm^3^ [26].

### 2.2. Patch-Based Data Preparation

The blood flow simulation generates pressure values on every face of the 3D artery mesh. However, FFR evaluation does not require pressure information at every point on the arterial wall; it depends only on the pressure along the artery centerline, which mimics the clinical procedure of inserting a pressure wire invasively to measure blood pressure at the center of the artery. Therefore, instead of using the full volumetric pressure field, only the average pressures along the centerline are extracted. Local 3D image patches are simultaneously extracted around centerline points. These patches capture imaging features of the surrounding vessel and are used as inputs to predict the corresponding centerline pressure, thereby linking anatomical imaging to hemodynamic function.

Furthermore, this strategy reduces data dimensionality, focuses on clinically relevant locations, and aligns the deep learning framework with real-world blood pressure measurement procedures.

For each point on the centerline $C_{n,m}$, where $m\in Z^{+}$ denotes the index of the centerline point in artery *n*, with volume origin ${\mathrm{VO}}_{n}\in R^{3}$ and voxel spacing $S_{n}\in R^{3}$, a 28×28×28 voxel patch $X\in R^{D\times H\times W}$ is extracted and centered at the point coordinates. The average pressure within a radius r=5mm around the centerline point is calculated and assigned as the label for the patch.

All coordinates are converted to local voxel coordinates relative to the artery centerline to ensure spatial consistency. Mathematically, the local voxel indices are computed as in Equation (1).

(1) $$\left(\begin{array}{c} i_{n,m}\\ j_{n,m}\\ k_{n,m} \end{array}\right)=\left(\left(\begin{array}{c} C_{n,m}^{x}\\ C_{n,m}^{y}\\ C_{n,m}^{z} \end{array}\right)-{\mathrm{VO}}_{n}\right)⊙S_{n},$$

where ⊙ denotes element-wise division.

A prerequisite for this computation is that volumes, centerlines, and pressure data are aligned within the same coordinate system to avoid spatial mismatch. This is ensured through a volume alignment procedure. Specifically, when a volume in the Right Anterior Superior (RAS) coordinate system is converted to a Left Posterior Superior (LPS) compliant format, the *x* and *y* coordinates are sign-flipped. After the conversion, the volume loses all spatial coordination. To align the converted volume, it must be translated from its new LPS coordinate center to the artery tree’s center and rotated by $-180^{\circ }$ along the x-axis if required. The volume center is computed as half of the element-wise product between the sum of the origin and spacing and the volume dimensions.

For each artery, the algorithm iterates along the centerline, extracting a 3D image patch centered at every point to capture local anatomical features. The corresponding pressure values are averaged within a small neighborhood around each centerline point to serve as the training label. All spatial coordinates are transformed into a local reference frame relative to the artery centerline, ensuring spatial consistency across arteries and patients.

This procedure systematically constructs a dataset of paired imaging patches and centerline pressure values, enabling the model to learn the mapping from anatomical structure to hemodynamic function. Consequently, the trained model can simulate blood pressure distributions and support non-invasive FFR estimation from coronary CT imaging.

### 2.3. Inverted Conditional Diffusion

The proposed ICD model is a modification of traditional conditional diffusion frameworks, in which the roles of input and label are repurposed. Unlike standard diffusion models that generate images conditioned on labels, ICD treats the labels (i.e., pressure values) as inputs and the anatomical features (image patches) as conditioning variables, effectively inverting the diffusion paradigm. This formulation enables the network to regress blood pressure values from local imaging features in a manner that conventional encoder-only networks cannot replicate.

#### 2.3.1. Forward Diffusion Process

The forward Markov chain is parameterized by a predefined variance schedule ${\left\{\beta_{t}\right\}}_{t=1}^{T}$, where $\beta_{t}$ denotes the noise variance at diffusion step *t*, and t∈[1,1000]. Let $y_{0}$ denote the simulated CFD blood pressure ground truth, and $y_{1:T}$ denote the sequence of latent variables. The forward process is defined as in Equations (2) and (3).

(2) $$q\left(y_{1:T}|y_{0}\right)=\prod_{t=1}^{T}q\left(y_{t}|y_{t-1}\right),$$

(3) $$q\left(y_{t}|y_{t-1}\right)=N\left(y_{t};\sqrt{1-\beta_{t}}\;y_{t-1},\beta_{t}I\right)$$

After *T* diffusion steps, the latent variable approaches an isotropic Gaussian distribution [26], as shown in Equation (4):

(4) $$q\left(y_{T}|y_{0}\right)\approx N\left(0,I\right).$$

#### 2.3.2. Reverse Diffusion Process

The reverse process learns a parameterized conditional distribution $p_{\theta }\left(y_{t-1}|y_{t},c\right)$, where *c* denotes the conditioning variable formed by concatenating imaging features and relative centerline coordinates. The model iteratively denoises $y_{T}$ back to the original pressure value $y_{0}$.

The reverse transitions are modeled as Gaussian distributions with learned mean $\mu_{\theta }$ and variance $\sigma_{t}^{2}$ following the DDPM sampling formulation [27], as shown in Equation (5).

(5) $$p_{\theta }\left(y_{t-1}|y_{t},c\right)=N(y_{t-1};\mu_{\theta }\left(y_{t},t,c\right),\sigma_{t}^{2}I).$$

The variance and mean are defined as in Equations (6) and (7).

(6) $$\sigma_{t}^{2}=\frac{1-{\bar{\alpha }}_{t-1}}{1-{\bar{\alpha }}_{t}}\;\beta_{t},$$

(7) $$\mu_{\theta }\left(y_{t},t,c\right)=\frac{1}{\sqrt{\alpha_{t}}}\left(y_{t}-\frac{1-\alpha_{t}}{\sqrt{1-{\bar{\alpha }}_{t}}}\epsilon_{\theta }\left(y_{t},t,c\right)\right),$$

where $\alpha_{t}=1-\beta_{t}$, ${\bar{\alpha }}_{t}=\prod_{s=1}^{t}\alpha_{s}$, and $\epsilon_{\theta }$ is a neural network trained to predict the added noise, as $\epsilon =\frac{y_{t}-\sqrt{{\bar{\alpha }}_{t}}y_{0}}{\sqrt{1-{\bar{\alpha }}_{t}}}$ [28].

Through iterative denoising, the model effectively reconstructs pressure values from imaging features by reversing the forward noise process.

#### 2.3.3. Conditioning and Sampling

Conditioning is implemented by concatenating the image patch features and relative centerline coordinates into the denoising network. During inference, the model initializes from Gaussian noise and iteratively applies the learned reverse transitions to recover predicted pressure values at centerline locations.

This strategy enables ICD to construct a latent representation from the pressure labels and propagate backward through the diffusion chain, allowing accurate regression even under complex non-linear anatomical–hemodynamic relationships. The complete ICD procedure is summarized in Algorithm 1.
**Algorithm 1** Proposed inverted conditional diffusion (ICD).1:**Input:** sample $y_{0}$, conditioning variables *c*, noise schedule ${\left\{\beta_{t}\right\}}_{t=1}^{T}$2:Define $\alpha_{t}=1-\beta_{t}$ and ${\bar{\alpha }}_{t}=\prod_{s=1}^{t}\alpha_{s}$3:**// Forward Process**4:**for** t=1 to *T* **do**5:      Sample ϵ∼N(0,I)6:      $y_{t}=\sqrt{{\bar{\alpha }}_{t}}\;y_{0}+\sqrt{1-{\bar{\alpha }}_{t}}\;\epsilon$7:**end for**8:**// Reverse Process**9:Train conditional network $\epsilon_{\theta }\left(y_{t},t,c\right)$ to predict ${\hat{\epsilon }}_{\theta }$10:**for** t=T down to 1 **do**11:      Sample z∼N(0,I) if t>1, else z=012:      Compute:$$y_{t-1}=\frac{1}{\sqrt{\alpha_{t}}}\left(y_{t}-\frac{\beta_{t}}{\sqrt{1-{\bar{\alpha }}_{t}}}\epsilon_{\theta }\left(y_{t},t,c\right)\right)+\sigma_{t}z$$13:**end for**14:**Ensure:** Denoised sample $y_{0}$

### 2.4. Architecture of ICD

**Figure 3:**
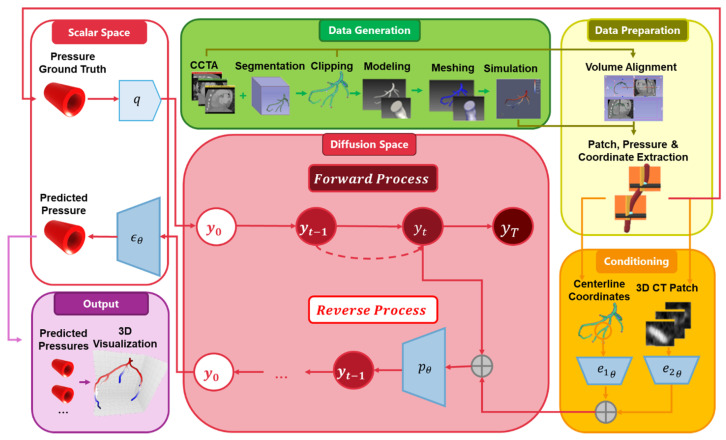
Architecture of the proposed ICD model for blood pressure prediction.

The architecture of the proposed ICD is shown in Figure 3. The ICD model is designed to predict blood pressure at each coronary artery centerline point by integrating local anatomical information with geometric positional cues.

To achieve this, the framework begins with a 3D convolutional encoder that processes a volumetric CCTA patch centered around the vessel lumen. This encoder consists of sequential 3D convolution, batch normalization, ReLU activation, and max-pooling layers, progressively extracting multi-scale spatial features. After the final convolutional block, the resulting feature map is flattened and projected into a compact 128-dimensional embedding that captures local vessel morphology, lumen intensity patterns, plaque burden, and surrounding tissue characteristics.

In parallel, each centerline coordinate $\left(C_{x},C_{y},C_{z}\right)$ is encoded through a lightweight linear mapping to produce a 3-dimensional geometric embedding. This embedding provides the model with structural context, enabling it to learn how blood pressure naturally varies along the artery according to vessel curvature, branch location, and proximity to stenotic regions. The anatomical and geometric embeddings are concatenated to form a unified 288-dimensional representation describing the imaging context and spatial identity of each target point. This fused representation serves as conditioning input for the diffusion process.

The diffusion module forms the core of the ICD model. Unlike conventional diffusion models used for image generation, our formulation inverts the process to operate directly in the regression domain. During training, Gaussian noise is progressively added to the ground truth pressure values, forming a forward diffusion trajectory. The model then learns the reverse denoising trajectory, where a neural network—conditioned on the fused anatomical–geometric representation—iteratively predicts a cleaner pressure estimate at each diffusion timestep. Two fully connected layers with ReLU activation serve as the denoising backbone, and a final linear layer outputs a single pressure value representing the blood pressure prediction at that centerline point. The ICD design eliminates sequential dependencies found in recurrent models and yields smooth, physically consistent pressure predictions that align more closely with coronary physiology.

### 2.5. Loss Function and Optimization

Huber loss is employed to optimize the proposed model. Huber loss outperforms other loss functions in time-series analysis by combining MSE and MAE, choosing MSE for small errors and MAE for large errors, providing more stable results [29]. The Huber loss function is defined as follows, where δ is a threshold parameter, *y* is the ground truth value, and $\hat{y}$ is the predicted value, as shown in Equation (8).

(8) $$L_{\delta }\left(y,\hat{y}\right)=\left\{\begin{array}{cc} \frac{1}{2}{\left(y-\hat{y}\right)}^{2}, & \mathrm{for}\;|y-\hat{y}|\leq \delta \\ \delta |y-\hat{y}|-\frac{1}{2}\delta^{2}, & \mathrm{otherwise} \end{array}\right.$$

The optimizer used is Adam with decoupled weight decay for its ability to optimize learning rate and weight decay simultaneously, reducing unnecessary hyperparameter tuning, speeding up convergence, and mitigating overfitting.

### 2.6. Evaluation Metrics

The evaluation metrics used in this study include $R^{2}$ score, Pearson correlation coefficient (PCC), normalized root mean squared error (NRMSE), and root mean squared error (RMSE).

$R^{2}$ Score: The coefficient of determination, which measures the proportion of variance in the dependent variable predictable from the independent variables, as shown in Equation (9).

(9) $$R^{2}=1-\frac{\sum_{i=1}^{m}{\left({\hat{y}}_{i}-y_{i}\right)}^{2}}{\sum_{i=1}^{m}{\left(\bar{y}-y_{i}\right)}^{2}}$$

Here, $y_{i}$ is the ground truth blood pressure for one patch, ${\hat{y}}_{i}$ is the predicted value, and $\bar{y}$ is the mean of the observed pressures.

Pearson Correlation Coefficient (PCC) measures the strength of the linear relationship between two continuous variables. Let ${\hat{y}}_{i}$ be the prediction, $\bar{\hat{y}}$ the mean of predictions, $y_{i}$ the ground truth, and $\bar{y}$ the mean of observations, as defined in Equation (10).

(10) $$\mathrm{PCC}=\frac{\sum_{i}\left({\hat{y}}_{i}-\bar{\hat{y}}\right)\left(y_{i}-\bar{y}\right)}{\sqrt{\sum_{i}{\left({\hat{y}}_{i}-\bar{\hat{y}}\right)}^{2}\sum_{i}{\left(y_{i}-\bar{y}\right)}^{2}}}$$

Root Mean Squared Error (RMSE) measures the average magnitude of prediction errors, as shown in Equation (11).

(11) $$\mathrm{RMSE}=\sqrt{\frac{1}{m}\sum_{i=1}^{m}{\left({\hat{y}}_{i}-y_{i}\right)}^{2}}$$

Normalized Root Mean Squared Error (NRMSE) normalizes RMSE relative to the observed mean, facilitating comparison across models with different scales, as dentoed in Equation (12).

(12) $$\mathrm{NRMSE}=\frac{\sqrt{\frac{1}{m}\sum_{i=1}^{m}{\left({\hat{y}}_{i}-y_{i}\right)}^{2}}}{\bar{o}}$$

Here, *m* is the number of samples, and $\bar{o}$ is the mean observed value.

### 2.7. Enrolled Datasets

Two datasets were used in this study: CCTA1000 and CCTA36. CCTA1000 corresponds to the publicly available ImageCAS dataset [30], which contains high-quality coronary CT angiography (CCTA) scans with expertly annotated coronary artery segmentations. CCTA36 is a private dataset [31] and includes patients with invasive blood ressure measurements.

The dataset consists of 3D CTA images captured by the Siemens 128-slice dual-source scanner from 1000 patients. For patients who had previously been diagnosed with coronary artery disease, early revascularization within 90 days after is included. The high-dose CTA is performed, and during the reconstruction, the 30–40% phase or the 60–70% phase is selected to obtain the best coronary artery images. The resulting scans have a spatial resolution of 512×512× (206–275) voxels, a planar resolution of 0.29–0.43 mm^2^, and spacing of 0.25–0.45 mm. The data was collected from realistic clinical cases at the Guangdong Provincial People’s Hospital during April 2012 to December 2018. Only the patients older than 18 years and with a documented medical history of ischemic stroke, transient ischemic attack or peripheral artery disease were eligible to be included. Finally, there were a total of 414 females and 586 males included, with the average ages being 59.98 and 57.68, respectively. The left and right coronary arteries in each image are independently labeled by two radiologists, and their results are cross-validated. The labeled coronary artery includes the left main coronary artery, left anterior descending coronary artery, left circumflex coronary artery, right coronary artery, diagonal 1, diagonal 2, diagonal 3, obtuse marginal branch 1, obtuse marginal branch 2, obtuse marginal branch 3, ramus intermedius, posterior descending arteries, acute marginal 1 and other blood vessels [30]. The data is made up of Nifti volume-labeled segmentations.

The CCTA36 dataset includes thirty-six patients with at least one coronary stenosis ≥50%. These cases were retrospectively collected, and each patient underwent SPECT MPI imaging followed by invasive FFR assessment, providing ground truth functional measurements.

For experiments, a subset of each dataset was used. From CCTA1000, 40 scans were used for training, 5 for validation, and 10 for testing. From CCTA36, 10 out of 36 patients were enrolled, with 6 scans used for training, 2 for validation, and 2 for testing. CCTA1000 is considered a medium-sized dataset, whereas CCTA36 represents a small, functionally annotated dataset.

## 3. Results

### 3.1. Quantitative Results of Blood Flow Prediction

To assess the performance of the proposed ResNet50-ICD model, it was compared to CNN-MLP, CNN-Attention-BiLSTM, CNN-ICD, ResNet50-MLP, and ResNet50-Attention-BiLSTM, all implemented in PyTorch 2.5 with a random seed of 42. The baseline architectures are as follows:

- CNN–MLP (CM) predicts blood pressure at each centerline point by jointly leveraging local image features and spatial geometry. A 3D CNN encoder extracts features from a cubic patch centered on the coronary artery using three convolutional blocks with batch normalization, ReLU activations, and max-pooling. The resulting feature map is flattened and projected into a 128-dimensional representation. Centerline coordinates are processed via a lightweight MLP into a 32-dimensional embedding. Both feature vectors are concatenated and passed through a two-layer regression head to produce the scalar pressure estimate.
- CNN–Attention-BiLSTM (CL) employs a CNN to extract 3D image features and integrates them with centerline coordinates, then applies a Bi-LSTM with attention to capture sequential dependencies along the vessel. The attended features are fed into a regression head.
- CNN–ICD (CD) is a variant where the ResNet in the proposed model is replaced with plain CNN layers.
- ResNet50–MLP (RM) replaces the CNN with a more powerful ResNet50 feature extractor while maintaining a simple regression head.
- ResNet50–Attention-BiLSTM (RL) replaces CNN layers in CNN-Att with ResNet-50 to extract deeper representations from patched CCTA images.

#### 3.1.1. CCTA1000 Case-Wise Evaluation

Using the same data splitting strategy, the proposed model was validated on the CCTA1000 dataset. Table 2 and Table 3 summarize the results.

#### 3.1.2. CCTA36 Case-Wise Evaluation

Table 4 and Table 5 summarize the evaluation metrics for the small CCTA36 dataset. Although the dataset is extremely limited and inconclusive, it provides some insight into model performance under constrained data conditions. The ResNet-based models each outperform in at least one metric, highlighting the data-intensive nature of diffusion models.

The results illustrate that the ResNet Inverse Conditional Diffusion (ICD) model achieved the highest average $R^{2}$ of 64.42%, RMSE of 0.974, and NRMSE of 0.154 on the medium-sized CCTA1000 dataset, with the second-highest Pearson correlation of 84.978. The CNN-ICD model achieved the second-best performance with an average $R^{2}$ of 61.093, RMSE of 0.984, and NRMSE of 0.157, ranking third in Pearson correlation at 84.656. These results demonstrate that the proposed ICD model achieves state-of-the-art performance in blood pressure prediction.

The performance suggests that the ICD model may also be effective for other non-linear regression and classification tasks. For small datasets, results were inconclusive, as different models excelled in isolated metrics. Due to order dependency, even without shuffling, each new branch carries the hidden output of the previous branch, which may reduce LSTM effectiveness.

The success of ICD is attributed to its order invariance and use of labels as input. By feeding the label as input, the model constructs a latent space from the label and works backwards, effectively teaching the network to derive underlying variables—a strategy that encoder-only models cannot achieve. Limitations include slower inference times and higher data requirements.

Finally, the dataset generation pipeline, including transformations and file conversions, enabled effective training by aligning different conditioning vectors within the same coordinate space. The running time of each model is presented in Table 6.

### 3.2. Qualitative Results of Blood Flow Prediction

**Figure 4:**
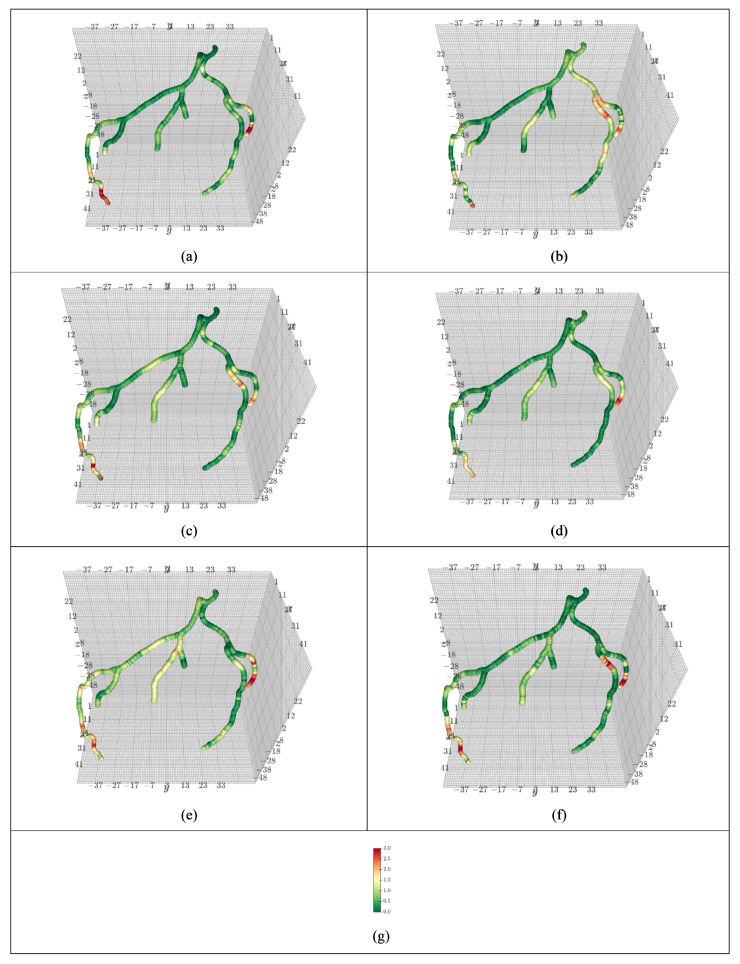
Average blood pressure prediction vs. label for one subject in the CCTA1000 dataset using (**a**) RD, (**b**) RL, (**c**) RM, (**d**) CD, (**e**) CL, and (**f**) CM models. The pseudo bar (**g**) indicates the absolute difference between the predicted blood pressure and the ground truth of the blood pressure generated during the CFD simulation in mmHg.

Figure 4 visualizes the absolute error between the label and the model prediction of coronary artery pressure at points on the centerline using one subject from the CCTA1000 dataset. Deep green symbolizes little to no error, yellow represents some error, and orange and red represent large error. The better a model performs on a given case, the fewer red spots will be visible. In Figure 4, the coronary artery stretches along the left anterior descending (LAD) sub-artery and left circumflex (LCX) sub-artery. The visualizations are housed in the LPS coordinate system. Most models struggle near sharp curvatures but RD (ours) has the lowest number of orange spots in that area, while keeping a green hue throughout the rest of the artery, which indicates little error. The closest competitor, CL, loses accuracy near the LCX. In Figure 4, the proposed diffusion models (a, d) were smoothest only having elevated error near caps.

**Figure 5:**
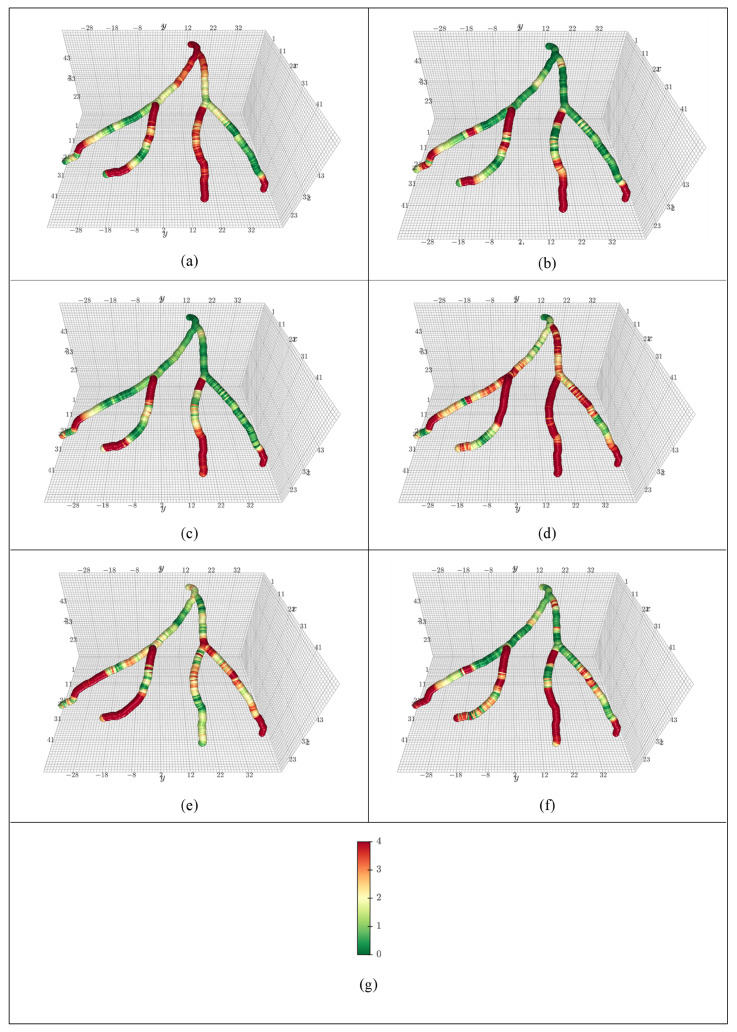
Average blood pressure prediction vs. label for one subject in the CCTA36 dataset using (**a**) RD, (**b**) RL, (**c**) RM, (**d**) CD, (**e**) CL, and (**f**) CM models. The pseudo bar (**g**) indicates the difference between the predicted blood pressure and the ground truth of the blood pressure generated during the CFD simulation in mmHg.

Figure 5 visualizes one testing subject in the CCTA36 dataset. RL and RM both perform well, being majorly green albeit with a number of red spots at the bifurcations. The attention of the LSTM enables it to maximize the little data available in the FFR dataset.

In Figure 5, the RL and CL models redeem themselves in the CCTA36 dataset by having more consistency along the major arteries, but every model struggles with the branched arteries. This is mostly due to the limited samples in the training dataset. A larger dataset would result in much better performance throughout.

**Figure 6:**
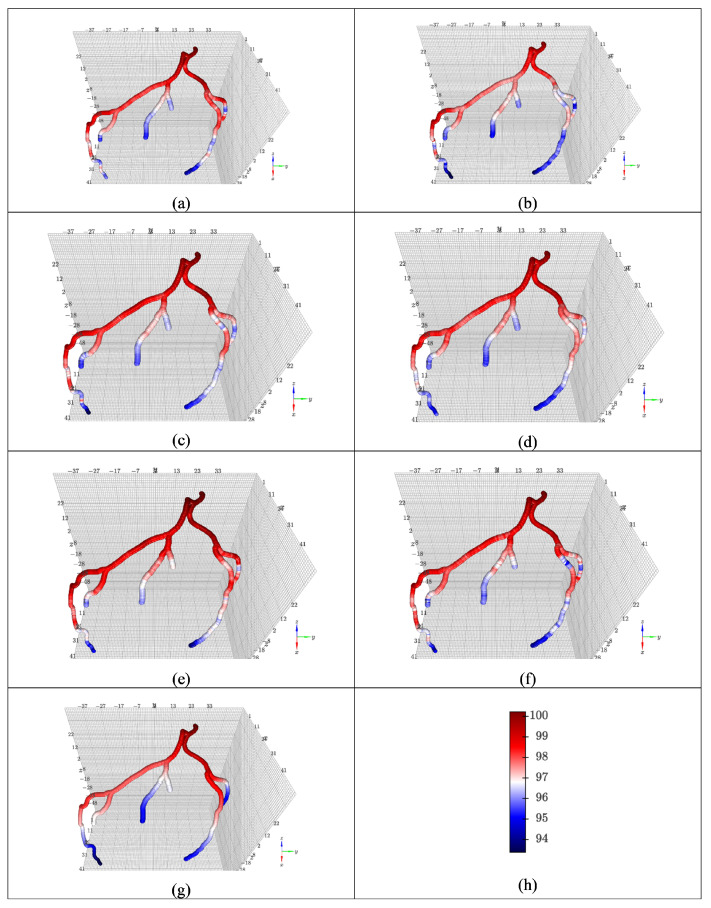
Average blood pressure for one subject in the CCTA1000 dataset using (**a**) RD, (**b**) RL, (**c**) RM, (**d**) CD, (**e**) CL, and (**f**) CM models, and (**g**) label. The pseudo bar (**h**) indicates the blood pressure in mmHg.

**Figure 7:**
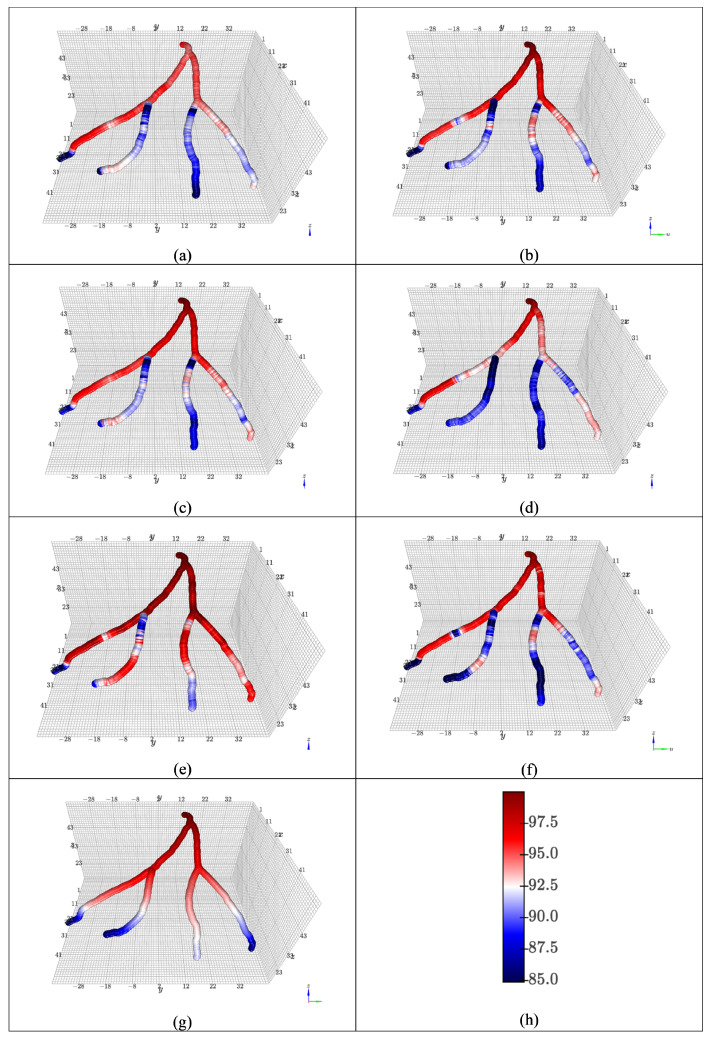
Average blood pressure for one subject in the CCTA36 dataset using (**a**) RD, (**b**) RL, (**c**) RM, (**d**) CD, (**e**) CL, and (**f**) CM models, and (**g**) label. The pseudo bar (**h**) indicates the blood pressure in mmHg.

The separated results of average mmHg pressure distribution prediction and label are shown in Figure 6 and Figure 7.

## 4. Discussion

The experimental results demonstrate that the proposed ResNet50-ICD model achieves superior performance on the medium-sized CCTA1000 dataset, consistently outperforming competing architectures in $R^{2}$, RMSE, and NRMSE, while maintaining a high Pearson correlation. The comparison across architectures highlights two important observations. First, deeper feature extraction using ResNet improves predictive stability compared with plain CNN-based encoders, suggesting that richer anatomical representation from CCTA patches is beneficial for pressure regression. Second, diffusion-based regression provides improved robustness over conventional MLP and BiLSTM regressors, likely due to its iterative refinement mechanism and reduced sensitivity to sequential ordering along the vessel centerline. In contrast, recurrent architectures such as BiLSTM may suffer from order dependency, where hidden-state propagation across branches can accumulate noise and degrade regression accuracy. These findings indicate that order-invariant modeling is advantageous for spatially structured coronary data.

From a clinical perspective, accurate and scalable pressure prediction is a critical step toward non-invasive FFR estimation. By avoiding computationally intensive CFD simulations, the proposed ICD framework substantially reduces inference complexity while preserving strong agreement with reference values. This efficiency may facilitate broader clinical deployment, particularly in scenarios requiring rapid decision-making or large-scale CCTA screening. However, performance on the small CCTA36 dataset was inconclusive, reflecting the data-intensive nature of diffusion-based models and the challenges of training high-capacity architectures with limited samples. Additional limitations include higher computational demands during training and the need for carefully standardized preprocessing pipelines. Future work will focus on improving data efficiency, incorporating multi-center datasets for better generalization, and exploring hybrid physics-informed learning strategies to further enhance robustness and clinical interpretability. In addition, fluid dynamical factors such as arterial wall shear stress, recirculating zone and pressure drops can be incorporated as features in the dataset for improved regression accuracy and physiologically realistic CFD modeling.

## 5. Conclusions

To summarize, to potentially support cardiologists in making accurate, non-invasive diagnoses in a timely manner, a novel pipeline and deep learning model were proposed. The deep regressor was a Scalar Diffusion Regressor with Resnet feature extraction. The pipeline was able to document and overcome many challenging obstacles to create a clean and compatible dataset, given only a CT scan and artery segmentation through patch and pressure extraction along a centerline after simulating blood flow and aligning volumes. The model was able to use this data pipeline to predict blood pressure for an entire artery with 64.42% R-squared, and left room for further model improvement. This study served as the foundation for future research in coronary artery disease detection as well as pushed the envelope further in the current landscape of bioinformatics.

## Figures and Tables

**Table 1:** Solver parameters employed to simulate blood flow using SimVascular.

Description	Value
Number of timesteps	150
Timestep size	0.001 s
Number of timesteps between restarts	10
Step construction	4
Residual criteria	0.001
SvLS type	GMRES
Tolerance on momentum equations	0.001
Tolerance on continuity equations	0.01
Tolerance on NS solver	0.01
Maximum iterations for SvLS NS solver	10
Maximum iterations for SvLS momentum loop	10

**Table 2:** R-squared and Pearson correlation of case-wise evaluation using subjects in CCTA1000.

Test Case	RD (Ours)		RL		RM		CD (Ours)		CL		CM	
	$R^{2}$	PCC	$R^{2}$	PCC	$R^{2}$	PCC	$R^{2}$	PCC	$R^{2}$	PCC	$R^{2}$	PCC
1	66.04	85.34	55.33	91.57	65.33	84.83	52.65	81.36	44.54	87.49	42.30	79.49
2	71.41	87.52	68.45	85.12	76.05	89.25	82.21	91.70	54.20	89.73	72.06	87.15
3	54.52	86.11	22.22	82.56	66.91	87.87	71.46	90.10	9.34	85.20	61.36	87.48
4	71.97	85.84	26.25	78.28	72.41	85.17	76.80	87.66	75.13	88.04	75.51	87.18
5	79.67	89.49	34.81	83.10	56.06	79.26	81.06	90.10	76.60	89.90	61.01	80.36
6	74.48	89.84	49.45	83.24	65.84	86.07	69.39	90.16	70.68	86.11	72.99	87.77
7	39.66	76.19	9.09	74.25	26.04	71.77	−7.27	60.17	50.83	75.18	−10.60	58.96
8	58.28	78.22	69.06	89.51	19.42	78.48	48.19	75.11	26.89	86.16	68.60	84.48
9	68.12	89.76	78.01	89.97	76.21	92.00	69.75	91.96	79.79	90.08	77.95	91.37
10	60.00	81.47	33.76	83.65	65.94	85.80	66.69	88.24	77.83	89.99	70.87	88.73
Mean	64.42	84.978	44.64	84.125	59.021	84.05	61.093	84.656	56.583	86.788	59.202	83.297

**Table 3:** RMSE and NRMSE (NRM) of case-wise evaluation using subjects in CCTA1000.

Test Case	RD (Ours)		RL		RM		CD (Ours)		CL		CM	
	RMSE	NRM	RMSE	NRM	RMSE	NRM	RMSE	NRM	RMSE	NRM	RMSE	NRM
1	0.81	0.14	0.93	0.15	0.82	0.14	0.96	0.16	1.03	0.17	1.05	0.17
2	0.86	0.13	0.91	0.13	0.79	0.12	0.68	0.10	1.09	0.16	0.85	0.13
3	0.81	0.18	1.06	0.23	0.69	0.15	0.64	0.14	1.15	0.25	0.75	1.15
4	1.06	0.14	1.72	0.23	1.05	0.14	0.96	0.13	1.00	0.14	0.99	0.14
5	0.80	0.12	1.43	0.21	1.18	0.18	0.77	0.12	0.86	0.13	1.11	0.17
6	0.79	0.14	1.11	0.20	0.91	0.16	0.86	0.15	0.85	0.15	0.81	0.14
7	1.21	0.21	1.48	0.26	1.33	0.23	1.61	0.28	1.09	0.19	1.63	0.29
8	0.89	0.17	0.77	0.14	1.24	0.23	0.99	0.19	1.18	0.22	0.77	0.14
9	1.23	0.15	1.03	0.12	1.07	0.13	1.20	0.14	0.98	0.12	1.03	0.12
10	1.28	0.17	1.65	0.22	1.18	0.16	1.17	0.16	0.95	0.13	1.09	0.15
Mean	0.974	0.155	1.209	0.189	1.026	0.164	0.984	0.157	1.018	0.166	1.008	0.161

**Table 4:** R-squared and Pearson correlation of case-wise evaluation using subjects in CCTA36.

Test Case	RD (Ours)		RL		RM		CD (Ours)		CL		CM	
	$R^{2}$	PCC	$R^{2}$	PCC	$R^{2}$	PCC	$R^{2}$	PCC	$R^{2}$	PCC	$R^{2}$	PCC
1	14.76	60.43	45.35	75.91	42.88	70.35	−25.50	53.16	22.59	73.92	11.64	74.61
2	31.85	81.49	21.49	49.61	22.93	53.86	21.18	54.70	12.77	46.36	32.09	59.03
Mean	23.305	70.96	33.42	62.76	32.905	62.105	−2.15	53.93	17.68	60.14	21.865	66.82

**Table 5:** RMSE and NRMSE (NRM) of case-wise evaluation using subjects in CCTA36.

Test Case	RD (Ours)		RL		RM		CD (Ours)		CL		CM	
	RMSE	NRM	RMSE	NRM	RMSE	NRM	RMSE	NRM	RMSE	NRM	RMSE	NRM
2	3.65	0.2413	2.92	0.1932	2.99	0.1975	4.43	0.2928	3.48	0.2300	3.72	0.2457
13	8.06	0.2008	8.65	0.2155	8.57	0.2135	8.67	0.2159	9.12	0.2272	8.04	0.2004
Mean	5.855	0.2211	5.785	0.2044	5.78	0.2055	6.55	0.2544	6.3	0.2286	5.88	0.2231

**Table 6:** Training timetable for 1000 epochs in seconds.

Dataset	RD (Ours)	RL	RM	CD (Ours)	CL	CM
CCTA1000	10,807.5	11,881.1	11,694.9	12,523.5	12,316.6	12,475.9
CCTA36	5016.11	4885.65	4811.25	5373.47	5446.84	5911.45

## Data Availability

The original data presented in the study are openly available at [30].

## Abbreviations

The following abbreviations are used in this manuscript:

CAD	Coronary Artery Disease
CCTA	Coronary Computed Tomography Angiography
CFD	Computational Fluid Dynamics
FFR	Fractional Flow Reserve
ICA	Invasive Coronary Angiography
PCI	Percutaneous Coronary Intervention
CABG	Coronary Artery Bypass Grafting
FFR_CT_	Fractional Flow Reserve Computed Tomography
LAD	Left Anterior Descending (artery)
RAS	Right Anterior Superior (coordinate system)
LPS	Left Posterior Superior (coordinate system)
GMRES	Generalized Minimal Residual Method
MLP	Multilayer Perceptron
LSTM	Long Short-Term Memory Network
Bi-LSTM	Bidirectional LSTM
PCC	Pearson Correlation Coefficient
RMSE	Root Mean Squared Error
NRMSE	Normalized Root Mean Squared Error
DDPM	Denoising Diffusion Probabilistic Model
ICD	Inverted Conditional Diffusion
PBDP	Patch-Based Dataset Pipeline
